# Supplementary material for: Thermoelectric Properties of Cotton Fabrics Dip-Coated in Pyrolytically Stripped Pyrograf® III Carbon Nanofiber Based Aqueous Inks
Source: Materials (Basel). 2023 Jun 12;16(12):4335. doi: 10.3390/ma16124335 (PMC10305365; doi:10.3390/ma16124335)
Supplement: Supplementary file 1 [file materials-16-04335-s001.zip › materials-2416466-supplementary.pdf]

Supporting Information

Thermoelectric Properties of Cotton Fabrics Dip-Coated in Pyrolytically Stripped Pyrograf® III Carbon Nanofiber Based Aqueous Inks

Antonio J. Paleo [1,*], Beate Krause [2], Maria F. Cerqueira [3,4], Jose M. González-Domínguez [5], Enrique Muñoz [6], Petra Pötschke [2] and Ana M. Rocha [1]

[1] 2C2T-Centre for Textile Science and Technology, University of Minho, Campus de Azurém, 4800-058 Guimarães, Portugal; amrocha@det.uminho.pt
[2] Leibniz-Institut für Polymerforschung Dresden e.V. (IPF), Hohe Str. 6, 01069 Dresden, Germany; krause-beate@ipfdd.de (B.K.); poe@ipfdd.de (P.P.)
[3] INL-International Iberian Nanotechnology Laboratory, Av. Mestre. Jose Veiga, 4715-330 Braga, Portugal; fcerqueira@fisica.uminho.pt
[4] CFUM–Center of Physics of the University of Minho, Campus de Gualtar, 4710-057 Braga, Portugal
[5] Instituto de Carboquímica ICB-CSIC, C/Miguel Luesma Castán 4, 50018 Zaragoza, Spain; jmgonzalez@icb.csic.es
[6] Facultad de Física, Pontificia Universidad Católica de Chile, Santiago 7820436, Chile; munoztavera@gmail.com
* Correspondence: ajpaleovieito@2c2t.uminho.pt

3.2. Raman analysis of as-received CNFs and dip-coated textiles

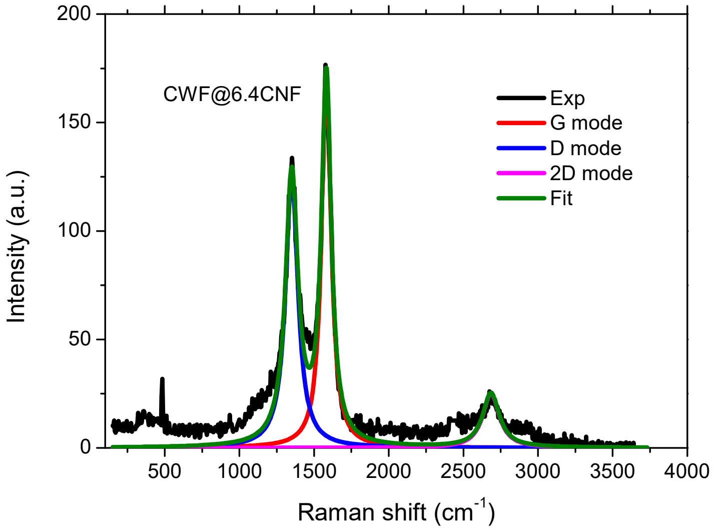

Figure S1. Example of the deconvolutions performed for parameters shown in Table 1.

3.3. XPS analysis of as-received CNFs and dip-coated textiles

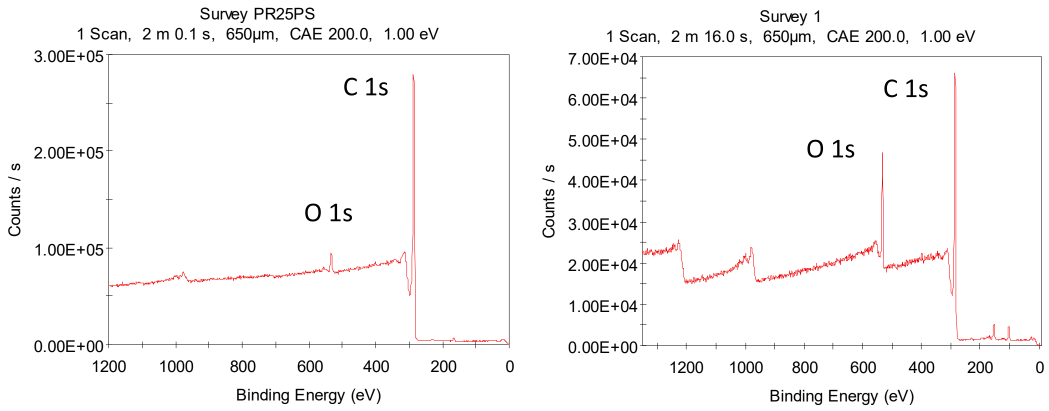

Figure S2. XPS survey spectra for CNFs (left) and the CWF@1.6CNF thermoelectric textile (right).

Table S1. XPS quantitative information extracted from the survey spectra displayed in Figure S1.

Sample	Composition (at%)				
	C	O	S	N	Si
**CNFs**	96	3	1	0	0
**CWF@1.6CNF**	78.1	15.9	0	1.6	4.4
